# Ethacridine Targets Bacterial Biofilms in Diabetic Foot Ulcers: A Multi-Target Mechanism Revealed by Network Pharmacology, Molecular Docking, Molecular Dynamics Simulation, and Clinical RT-qPCR Validation

**DOI:** 10.3390/cimb47100870

**Published:** 2025-10-21

**Authors:** Tianbo Li, Yuming Zhuang, Jiangning Wang, Lei Gao

**Affiliations:** 1 Plastic Surgery Department, Capital Medical University Affiliated Beijing Shijitan Hospital, Beijing 100038, China; litianbo3285@bjsjth.cn (T.L.); wangjn@bjsjth.cn (J.W.); 2 School of Traditional Chinese Medicine, Capital Medical University, Beijing 100000, China; zhuangyuming@mail.ccmu.edu.cn

**Keywords:** ethacridine, diabetic foot ulcers, antibacterial, network pharmacology, molecular docking, molecular dynamics simulation

## Abstract

Objective: This study aimed to systematically investigate the potential antibacterial mechanisms of ethacridine in the treatment of diabetic foot ulcers (DFUs) by integrating network pharmacology, molecular docking, and molecular dynamics simulation approaches. Methods: The potential targets of ethacridine were predicted using the SwissTargetPrediction and PharmMapper databases and subsequently converted to gene symbols via the UniProt database. DFU-related and antibacterial-related targets were retrieved from the GeneCards and OMIM databases. The overlapping targets among ethacridine, DFU, and antibacterial-related genes were identified as candidate therapeutic targets. A “drug–disease–target” network was constructed using Cytoscape, while protein–protein interaction (PPI) networks were built through the STRING database. GO and KEGG enrichment analyses were performed using R software. Molecular docking was conducted to evaluate the binding affinities between core compounds and hub targets. Furthermore, molecular dynamics (MD) simulation was applied to assess the binding stability of the top-ranked compound–target complex. Finally, RT-qPCR was conducted on wound edge tissue samples from DFU patients treated with ethacridine to experimentally validate the mRNA expression of predicted hub genes. Results: A total of 302 potential ethacridine-related targets, 4264 DFU-related targets, and 1942 antibacterial-related targets were identified. Intersection analysis revealed 105 common targets potentially involved in the antibacterial effects of ethacridine against DFU. PPI network analysis highlighted 10 hub targets, including *AKT1, EGFR, SRC, HSP90AA1,* and *MMP9*. GO enrichment indicated significant involvement in responses to reactive oxygen species, regulation of inflammatory responses, responses to lipopolysaccharide, and bacterial molecular patterns. KEGG pathway analysis identified 157 relevant pathways, including the lipid and atherosclerosis, TNF signaling, IL-17 signaling, and the AGE–RAGE signaling pathways in diabetic complications. Molecular docking demonstrated favorable binding affinities (all < −5.0 kcal/mol) between ethacridine and the hub targets, with the strongest binding observed between *MMP9* and ethacridine (−9.8 kcal/mol). These docking results suggest possible interaction tendencies that may contribute indirectly to Ethacridine’s network-level regulatory effects, rather than direct binding to all targets in vivo. Molecular dynamics simulation further confirmed the stable interaction between *MMP9* and ethacridine. RT-qPCR validation in clinical DFU tissue samples demonstrated expression trends of key genes consistent with in silico predictions. These results reflect transcriptional regulation consistent with pathway modulation predicted by the network analysis, rather than direct protein–ligand binding across all targets. Conclusion: Ethacridine may exert antibacterial effects against bacterial biofilms in DFU through multi-target and multi-pathway mechanisms. These findings highlight ethacridine’s translational potential as a safe, readily available, and mechanistically validated topical agent for the clinical management of biofilm-associated diabetic foot infections.

## 1. Introduction

Diabetic foot ulcers (DFUs) are one of the most devastating and costly complications of diabetes mellitus, characterized by chronic, non-healing wounds that significantly increase the risk of infection, lower-limb amputation, and mortality [1]. The persistent presence of polymicrobial infection and the formation of bacterial biofilms on the wound surface are major impediments to effective healing and represent key challenges in DFUs management [2,3]. “Although current DFU management combines debridement, infection control, revascularization, and advanced dressings, these measures often fail to eradicate biofilm-associated infections or restore normal healing dynamics. Conventional antibiotics have limited penetration into biofilms and are increasingly compromised by antimicrobial resistance, while topical antiseptics may exhibit cytotoxicity or inadequate efficacy in chronic wounds. Consequently, there remains an urgent need for safe, affordable agents that can simultaneously disrupt biofilms, modulate inflammation, and promote tissue regeneration—properties that make ethacridine a compelling therapeutic candidate.” [4].

Ethacridine, an acridine derivative, is a well-known topical antiseptic with broad-spectrum antibacterial activity. It has been historically used in wound disinfection due to its ability to disrupt microbial membranes and interfere with bacterial replication [5]. Recent studies suggest that ethacridine may also inhibit biofilm formation and exert immunomodulatory effects, making it a promising candidate for treating DFUs complicated by biofilm-associated infections [6]. However, the precise molecular mechanisms underlying its antibacterial actions in the context of DFUs remain unclear.

In recent years, network pharmacology has emerged as a powerful tool to systematically elucidate the multi-target and multi-pathway mechanisms of bioactive compounds in complex diseases [7]. By integrating pharmacological target prediction, disease-related gene mapping, and pathway enrichment analyses, network pharmacology offers a holistic framework to decipher drug-disease interactions. Complementing this approach, molecular docking and molecular dynamics (MD) simulations provide atomic-level insights into the binding affinity and structural stability of drug-target interactions, enhancing the predictive accuracy of computational modeling [8,9].

In this study, we employed an integrative approach combining network pharmacology, molecular docking, and MD simulations to systematically explore the antibacterial mechanisms of ethacridine in the treatment of DFU. Additionally, the expression levels of key hub genes were validated using RT-qPCR in wound edge tissue samples from DFU patients treated with ethacridine, thereby bridging computational predictions with clinical evidence. Our findings aim to provide new insights into the therapeutic potential and mechanistic basis of ethacridine as a multi-target antibacterial agent in DFU management.

## 2. Materials and Methods

### 2.1. Active Compound and Target Identification

The chemical structure of ethacridine was retrieved from the PubChem database (https://pubchem.ncbi.nlm.nih.gov, accessed on 12 June 2025) and its SMILES notation was obtained. Potential protein targets of ethacridine were predicted using two established pharmacoinformatics platforms: SwissTargetPrediction (http://www.swisstargetprediction.ch, accessed on 12 June 2025) and PharmMapper (www.lilab-ecust.cn/pharmmapper/, accessed on 12 June 2025). To ensure biological relevance and data accuracy, only targets classified as “Reviewed” and of “Homo sapiens” origin were retained. These targets were then annotated and standardized using the UniProt database (https://www.uniprot.org, accessed on 12 June 2025), and duplicates were removed to generate a refined list of ethacridine-related targets.

### 2.2. Prediction of Potential Targets of Ethacridine Against Diabetic Foot Ulcers and Antibacterial Activity

The disease-related targets for “diabetic foot ulcer” and “antimicrobial” were retrieved separately using the GeneCards database (https://www.genecards.org, accessed on 12 June 2025) and the Online Mendelian Inheritance in Man (OMIM) database (https://www.omim.org, accessed on 12 June 2025). All retrieved targets were compiled, and duplicates were removed. The intersection of three target sets—ethacridine-related targets, diabetic foot ulcer-related targets, and antimicrobial-related targets—was determined to identify potential therapeutic targets of ethacridine in the treatment of DFU with antibacterial indications. The overlapping targets were visualized using an online Venn diagram tool ((https://bioinformatics.psb.ugent.be/webtools/Venn/, accessed on 12 June 2025).

### 2.3. Construction and Analysis of the Ethacridine–Disease Target Network

Information on ethacridine and its intersecting targets was organized using Microsoft Excel to generate input files, including the “Network” and “Type” files. These data were then imported into Cytoscape software (version 3.7.2) to construct and visualize the interaction network between ethacridine and its potential therapeutic targets for diabetic foot ulcer and antibacterial activity.

### 2.4. Protein–Protein Interaction (PPI) Network Construction and Analysis

The intersecting targets of ethacridine related to both diabetic foot ulcer and antimicrobial activity were imported into the STRING database (https://string-db.org/, accessed on 12 June 2025) to construct a protein–protein interaction (PPI) network. The species was set to “Homo sapiens” and the minimum required interaction score was set to the default medium confidence. The resulting PPI network was exported and further analyzed using Cytoscape software (version 3.7.2). The CytoNCA plugin (version 2.1.6) was employed to perform topological analysis of the network, enabling the identification of hub targets based on centrality metrics.

### 2.5. GO and KEGG Pathway Enrichment Analysis

Gene Ontology (GO) and Kyoto Encyclopedia of Genes and Genomes (KEGG) pathway enrichment analyses were performed on the potential therapeutic targets of ethacridine against diabetic foot ulcers and bacterial infection using R software (version 4.4.2). The R (version 4.4.2) packages “DOSE (version 4.2.0),” “clusterProfiler (version 4.14.3),” “enrichplot (version 1.20.0),” and “enrichKEGG (version 4.14.3),” available through the BiocManager repository, were employed for the analysis. Enriched GO terms and KEGG pathways with a *p*-value < 0.05 were considered statistically significant. Visualization of enrichment results was carried out using Bioinformatics online platform (http://www.bioinformatics.com.cn, accessed on 12 June 2025).

### 2.6. Molecular Docking Analysis

Molecular docking is a widely used computational technique in drug discovery for accurately predicting the binding mode and affinity of small-molecule ligands with known protein structures. To further evaluate the reliability of key compounds and core targets associated with the antibacterial effect of ethacridine in the treatment of diabetic foot ulcers, molecular docking analysis was performed to assess the binding interactions between ethacridine and its predicted protein targets.

The 2D structure of ethacridine was obtained from the PubChem database (https://pubchem.ncbi.nlm.nih.gov/, accessed on 12 June 2025) and converted into a 3D conformation with minimized free energy using ChemBio3D Ultra 14.0 software. Core human receptor proteins corresponding to the predicted targets were identified using the UniProt database (https://www.uniprot.org/, accessed on 12 June 2025) with the filter set to “Homo sapiens.” The 3D crystal structures of these receptor proteins were then downloaded from the Protein Data Bank (PDB, https://www.rcsb.org/, accessed on 12 June 2025).

Prior to docking, receptor proteins were prepared by removing water molecules and original ligands using PyMOL software (version 2.3.4). Hydrogen atoms were added using AutoDockTools (version 1.5.6). The active binding sites of the receptors were defined using the Grid Box function in AutoDockTools (version 1.5.6). Molecular docking was performed with AutoDock Vina (v1.1.2). Protein structures were prepared by removing crystallographic waters and co-ligands in PyMOL, adding polar hydrogens and Gasteiger charges in AutoDockTools (v1.5.6). For each target, the search grid was centered on the known active site (defined by the co-crystallized ligand or canonical catalytic pocket) with a cubic box of 24 × 24 × 24 Å; grid spacing used Vina defaults. Docking settings were as follows: exhaustiveness = 16, num_modes = 20, energy_range = 4 kcal·mol^−1^. Poses with Vina score ≤ −5.0 kcal·mol^−1^ were retained for analysis, and the lowest-energy pose per target was used for visualization. Binding energies reported in the Results Section 3 are Vina scores.

### 2.7. Molecular Dynamics Simulation

Molecular dynamics (MD) simulations were conducted using GROMACS version 2022 to evaluate the dynamic stability of the optimal protein–ligand complexes obtained from molecular docking. Force field parameters were generated with the pdb2gmx tool in GROMACS and supplemented with ligand topology files from the Automated Force Field (AutoFF) web server (https://cloud.hzwtech.com/web/product-service?id=36, accessed 12 June 2025). During simulations, the CHARMM36 force field was applied to protein molecules and CHARMM General Force Field (CGenFF) parameters to the ligands.

Each complex was placed in a triclinic TIP3P water box with a buffer distance of ≥1.0 nm around the solute and neutralized with counter-ions using gmx genion. Long-range electrostatics were treated with the Particle Mesh Ewald (PME) method (real-space cutoff = 1.0 nm), and Lennard-Jones interactions used the same cutoff. Periodic boundary conditions were applied in all directions. All covalent bonds involving hydrogens were constrained with the LINCS algorithm (order 4), and the Verlet leap-frog integrator was used with a 2 fs integration time step (energy stability was verified with 1–2 fs during setup; 2 fs was used for production).

Energy minimization comprised 3000 steps of steepest descent followed by 2000 steps of conjugate-gradient optimization to remove steric clashes. Minimization proceeded in three stages: (1) water molecules with solute restrained, (2) counter-ions with solute restrained, and (3) the entire system without restraints. After minimization, systems were equilibrated under NVT (1 ns) using the v-rescale thermostat (T = 310 K, τT = 0.1 ps) and then NPT (1 ns) using the Parrinello–Rahman barostat (P = 1 bar, τP = 2.0 ps), with heavy-atom positional restraints (k = 1000 kJ·mol^−1^·nm^−2^) applied during equilibration and released for production.

Production trajectories were run for 100 ns in the NPT ensemble at 310 K and 1 bar, saving coordinates every 10 ps. System stability and conformational behavior were analyzed using built-in GROMACS tools: gmx rms (RMSD), gmx rmsf (RMSF), gmx gyrate (radius of gyration), gmx sasa (solvent-accessible surface area), and gmx hbond (hydrogen-bond analysis).

### 2.8. RT-qPCR Experiment

To further verify whether the expression of predicted biomarkers in diabetic foot ulcer (DFU) tissues was consistent with the results of bioinformatics analysis, we collected wound edge tissue samples from 10 DFU patients treated with ethacridine and 10 non-ethacridine treated DFU control patients at Capital Medical University Affiliated Beijing Shijitan Hospital. The relative mRNA expression levels of core genes were assessed using RT-qPCR. All participants provided written informed consent prior to enrollment, and the study protocol was approved by the Ethics Committee of Capital Medical University Affiliated Beijing Shijitan Hospital (approval code: IIT2025-002-001, approval date: 10 March 2025). RNA was extracted from the samples using TRIzol reagent (Invitrogen, 15596018, Carlsbad, CA, USA); reverse transcription was performed using the All-in-One RT MasterMix kit (Life-iLab, AN33L719, Shanghai, China), and primers were synthesized by Shanghai Shengong Biotech Co., Ltd. (Shanghai, China) The expression levels of genes were calculated using the 2^−ΔΔCt^ method, with GAPDH as the reference gene, and the differences were analyzed using Student’s *t*-test in GraphPad Prism 10 software (*p* < 0.05). The experimental reaction system, conditions, and related primer sequences are referred to in Table 1, Table 2, Table 3, Table 4 and Table 5.

### 2.9. Statistical Analysis

All statistical procedures were conducted using R software (version 4.4.2). For comparisons between two independent groups, the Wilcoxon rank-sum test was applied, with *p* values less than 0.05 considered statistically significant. In the RT-qPCR experiments, differences in gene expression levels were analyzed using the *t*-test, with a significance threshold set at *p* < 0.05.

## 3. Result

### 3.1. Identification of Active Compound and Related Targets of Ethacridine

To identify the pharmacologically relevant targets of ethacridine, its canonical SMILES structure (CCOC1=CC2=C(C3=C(C=C(C=C3)N)N=C2C=C1)N) was retrieved from the PubChem database (https://pubchem.ncbi.nlm.nih.gov/, accessed on 12 June 2025). The SMILES string was then used as input for target prediction via two widely validated online platforms: Swiss Target Prediction (http://www.swisstargetprediction.ch/, accessed on 12 June 2025) and PharmMapper (www.lilab-ecust.cn/pharmmapper/, accessed on 12 June 2025). The predicted targets from both databases were subsequently standardized and annotated using the UniProt database (https://www.uniprot.org/, accessed on 12 June 2025), with the filters set to retain only targets marked as “Reviewed” and derived from Homo sapiens. After integrating and deduplicating the results from both sources, a total of 302 non-redundant ethacridine-related protein targets were identified for further analysis.

### 3.2. Identification of Common Targets Among Ethacridine, Diabetic Foot Ulcer, and Antibacterial Gene Sets

**Figure 1:**
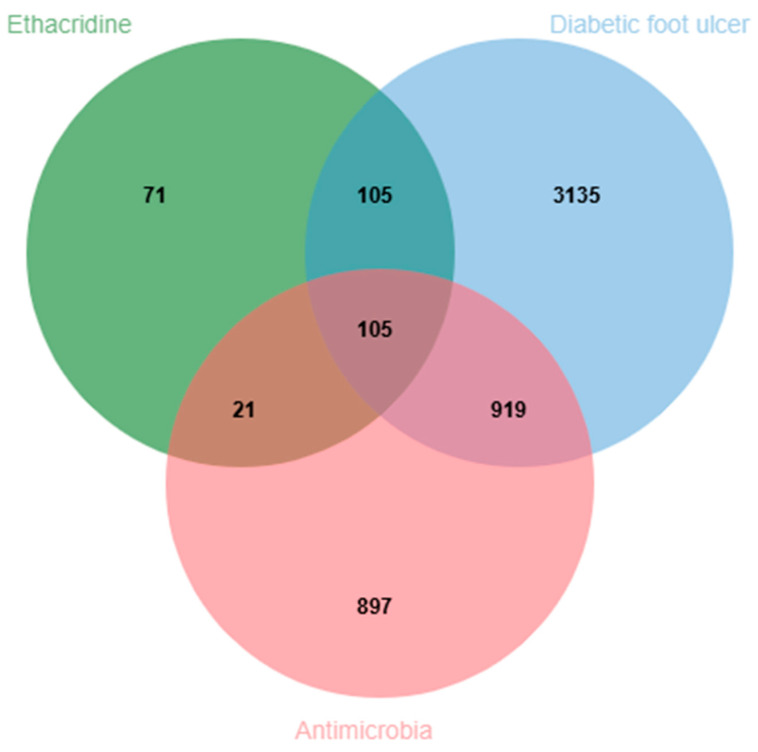
Venn diagram of ethacridine-associated targets, diabetic foot ulcer (DFU)-related targets, and antibacterial-related targets.

To identify disease-related targets relevant to diabetic foot ulcer (DFU) and antibacterial activity, two keyword-based searches were conducted using the terms “diabetic foot ulcer” and “antimicrobial” in both the GeneCards (https://www.genecards.org/, accessed on 12 June 2025) and Online Mendelian Inheritance in Man (OMIM) (https://www.omim.org, accessed on 12 June 2025) databases. After data integration and removal of duplicate entries, a total of 4264 DFU-related targets and 1942 antibacterial-related targets were obtained. To explore the potential therapeutic relevance of ethacridine in DFU with bacterial infection, an intersection analysis was performed among the previously identified 302 ethacridine-related targets, the DFU-related targets, and the antibacterial-related targets. This yielded a total of 105 overlapping targets, which were considered the putative core targets of ethacridine in treating DFU through antibacterial mechanisms. The intersection of these three target sets is illustrated in Figure 1.

### 3.3. Compound–Target–Disease Network of Ethacridine in Diabetic Foot Ulcer and Antibacterial Contexts

**Figure 2:**
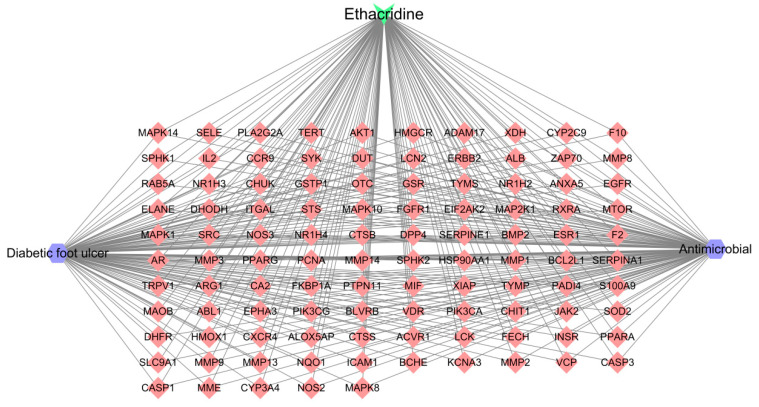
Network diagram of ethacridine and its potential targets related to diabetic foot ulcer and antibacterial activity. Green node: Ethacridine; Blue hexagons: Disease or pharmacological terms (Diabetic foot ulcer, Antimicrobial); Red diamonds: Target genes/proteins.

To visualize the interaction between ethacridine and its potential therapeutic targets for diabetic foot ulcer and antibacterial activity, a compound–target–disease network was constructed using Cytoscape version 3.7.2. The network included ethacridine as the active compound and 105 intersecting targets identified in the previous analysis. As shown in Figure 2, the network consisted of 108 nodes and 315 edges, where nodes represent chemical components, disease targets, or disease entities, and edges indicate predicted associations between them. In the network, squares denote the compound (ethacridine), diamonds represent the predicted disease-related targets, and hexagons correspond to the disease components (diabetic foot ulcer and bacterial infection). The structure of the network suggests a multi-target, multi-pathway interaction model, indicating that ethacridine may exert its therapeutic effects via modulation of multiple genes and pathways associated with DFU and microbial pathogenesis.

### 3.4. PPI Network Construction and Identification of Hub Targets

**Figure 3:**
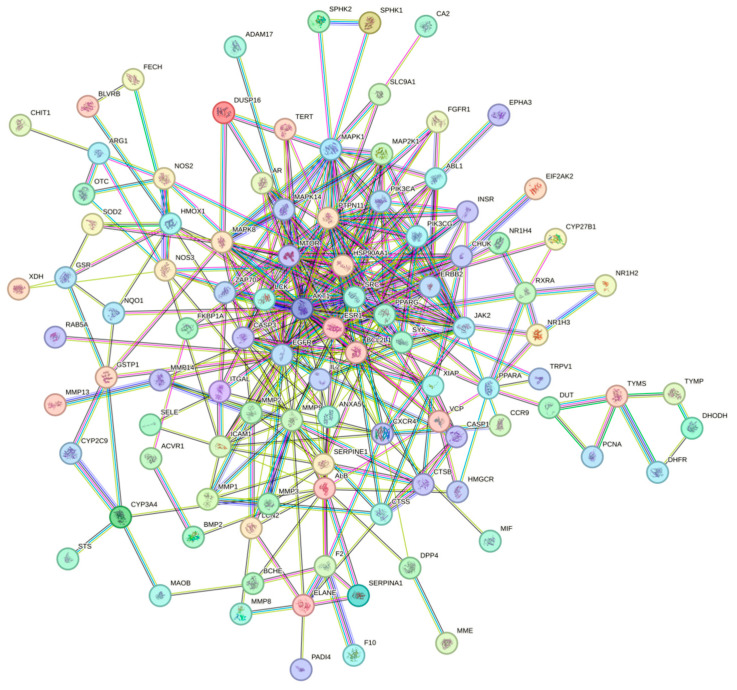
Protein–protein interaction (PPI) network of potential targets of ethacridine in the treatment of diabetic foot ulcer with antibacterial relevance. The different node colors in this figure represent distinct functional clusters generated by the STRING database, indicating groups of proteins involved in similar biological processes or pathways.

To investigate the interactions among the potential therapeutic targets of ethacridine in the treatment of diabetic foot ulcer (DFU) with antibacterial relevance, a total of 105 predicted targets were imported into the STRING database (https://string-db.org/, accessed on 12 June 2025). The species was set to Homo sapiens, with the minimum required interaction score set to 0.7 (high confidence). Disconnected nodes were hidden, and all other parameters were left as default. The resulting protein–protein interaction (PPI) network comprised 102 nodes and 352 edges, as illustrated in Figure 3.

**Figure 4:**
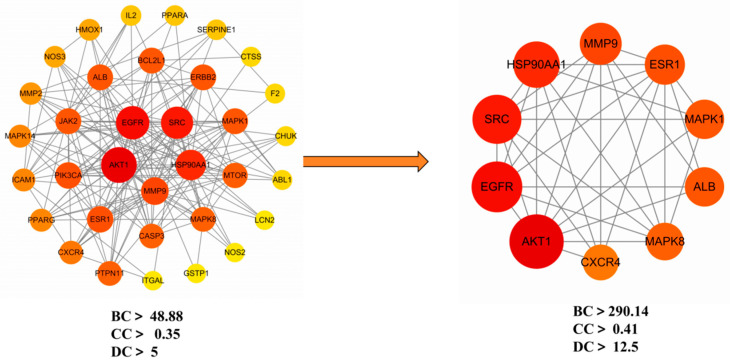
Identification of hub targets in the PPI network of ethacridine against diabetic foot ulcer and bacterial infection. The color gradient from yellow to red represents increasing node centrality values (BC, CC, DC), with darker red nodes indicating higher importance within the network.

Subsequently, the interaction network was exported and analyzed in Cytoscape version 3.7.2. The CytoNCA plugin was used to perform topological analysis based on three centrality parameters: Betweenness Centrality (BC), Closeness Centrality (CC), and Degree Centrality (DC). A two-step filtering strategy was applied to screen hub genes. As a result, 10 hub targets were identified and visualized in Figure 4, with detailed topological scores summarized in Table 6.

The top-ranking targets by degree value were: *AKT1, EGFR, SRC, HSP90AA1, MMP9, ESR1, MAPK1, ALB, MAPK8,* and *CXCR4*. These genes occupy central positions within the interaction network and are likely to play pivotal roles in the antibacterial mechanisms of ethacridine in DFU treatment.

### 3.5. GO Enrichment Analysis of Potential Targets

To investigate the biological functions of the 105 potential targets of ethacridine in the treatment of diabetic foot ulcer (DFU) with antibacterial activity, Gene Ontology (GO) enrichment analysis was performed using the R packages clusterProfiler, org.Hs.eg.db, enrichplot, and ggplot2. The analysis covered three GO categories: biological process (BP), molecular function (MF), and cellular component (CC). Enrichment results with *p* < 0.05 were considered statistically significant.

In the biological process (BP) category, a total of 1901 enriched terms were identified. These were primarily associated with cellular responses to chemical stress, reactive oxygen species (ROS), regulation of inflammatory responses, response to lipopolysaccharide (LPS), response to molecules of bacterial origin, oxidative stress, nutrient levels, and exogenous biological stimuli.

In the molecular function (MF) category, 150 enriched terms were obtained, mainly involving transmembrane receptor protein kinase activity, transmembrane receptor protein tyrosine kinase activity, protein tyrosine kinase activity, carboxylic acid binding, nuclear receptor activity, ligand-activated transcription factor activity, organic acid binding, serine-type endopeptidase activity, and serine-type peptidase activity.

In the cellular component (CC) category, 79 terms were significantly enriched, with the most prominent locations being membrane rafts, membrane microdomains, vesicle lumens, secretory granule lumens, cytoplasmic vesicle lumens, plasma membrane rafts, caveolae, and focal adhesions.

**Figure 5:**
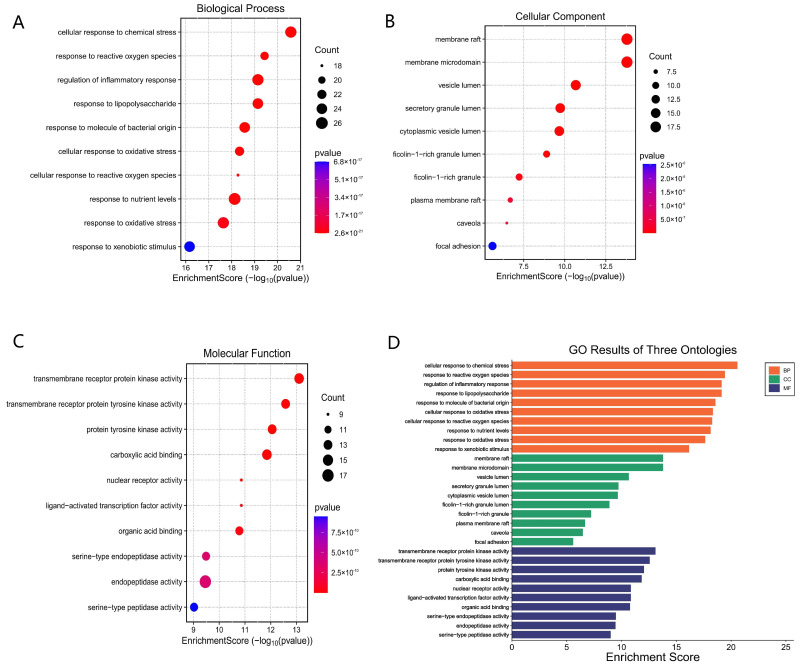
GO functional enrichment analysis of potential therapeutic targets of ethacridine in the treatment of diabetic foot ulcer and bacterial infection. (**A**) Bubble plot of biological process (BP) enrichment. (**B**) Bubble plot of cellular component (CC) enrichment. (**C**) Bubble plot of molecular function (MF) enrichment. (**D**) Bar chart summarizing the top enriched terms from BP, CC, and MF categories. Dot size represents the number of enriched genes, and color intensity indicates the statistical significance (adjusted *p*-value).

The top 10 significantly enriched terms from each GO category were visualized using bubble plots and bar plots generated via the Microbioinformatics platform. The results are presented in Figure 5.

### 3.6. KEGG Pathway Enrichment Analysis

To further elucidate the biological mechanisms underlying the potential therapeutic effects of ethacridine in diabetic foot ulcer (DFU) with antibacterial indications, Kyoto Encyclopedia of Genes and Genomes (KEGG) pathway enrichment analysis was performed on the 105 identified targets using the clusterProfiler R package in conjunction with the pathview library and the enrichKEGG function. Enriched pathways with *p* < 0.05 were considered statistically significant.

A total of 157 significantly enriched pathways were identified. These pathways were primarily associated with lipid and atherosclerosis, the tumor necrosis factor (TNF) signaling pathway, endocrine resistance, relaxin signaling pathway, ferroptosis, the interleukin-17 (IL-17) signaling pathway, and the advanced glycation end-products–receptor for AGEs (AGE–RAGE) signaling pathway in diabetic complications.

**Figure 6:**
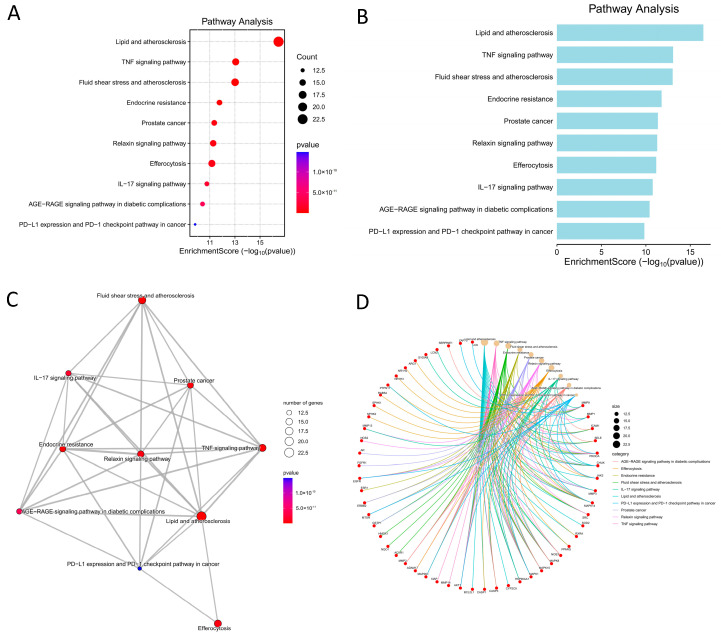
KEGG pathway enrichment analysis of potential therapeutic targets of ethacridine in the treatment of diabetic foot ulcer and bacterial infection. (**A**) Bubble plot of the top 10 significantly enriched KEGG pathways. The size of each bubble represents the number of involved genes, while the color gradient reflects the adjusted *p*-value. (**B**) Bar chart of the top 10 KEGG pathways ranked by statistical significance, showing the number of enriched genes per pathway. (**C**) Pathway–pathway interaction network illustrating the functional relationships among enriched KEGG pathways based on shared gene components. (**D**) Chord diagram of pathway–target interactions, showing the connections between core KEGG pathways and their associated gene targets.

The top 10 enriched pathways, ranked by *p*-value, were visualized using a bubble plot, as shown in Figure 6. Detailed information on these pathways and associated genes is presented in Table 7.

### 3.7. Molecular Docking Validation Between Core Targets and Ethacridine

Binding affinity is considered a key indicator for assessing the stability and strength of interactions between active compounds and their protein targets. To evaluate the potential binding between ethacridine and the identified hub proteins, molecular docking analysis was performed on the top 10 core targets, including *AKT1, EGFR, SRC, HSP90AA1, MMP9, ESR1, MAPK1, ALB, MAPK8,* and *CXCR4*.

As shown in Table 8, the minimum binding energies of ethacridine with all 10 proteins were less than −5.0 kcal/mol, indicating theoretically favorable binding affinity and structural compatibility under in silico conditions. In this study, the purpose of molecular docking and molecular dynamics simulations was to predict potential binding sites and binding trends between Ethacridine and its putative protein targets. These results reflect only theoretical binding potential and do not demonstrate actual binding under in vivo or in vitro conditions.

Among these interactions, MMP9 exhibited the strongest binding affinity, with a minimum binding energy of −9.8 kcal/mol, suggesting that MMP9 may serve as a critical antibacterial target in DFU and a key molecular target of ethacridine.

**Figure 7:**
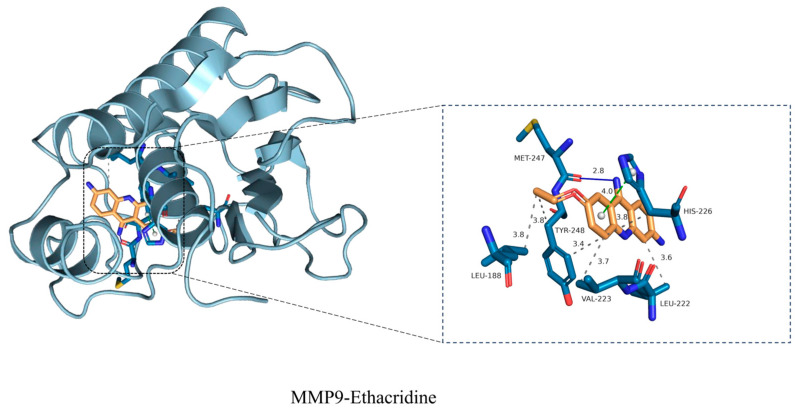
Docking pose of Ethacridine in the MMP-9 catalytic domain. Left, the protein structure (MMP9) is shown in light blue cartoon style, overall cartoon showing Ethacridine (orange) bound in the substrate-binding cleft. Right, zoom-in of the binding pocket highlighting key residues (LEU-188, HIS-226, LEU-222, VAL-223, MET-247, TYR-248). The key interacting amino acid residues are displayed in blue sticks. Dashed lines indicate predicted polar/hydrophobic contacts; numbers denote contact distances in Å. The pose corresponds to the top-ranked docking solution; distances were measured from the docking output.

Ethacridine docked into the substrate-binding cleft of MMP-9 (Figure 7), forming short polar contacts with HIS-226 and TYR-248 (≈2.8–3.4 Å) and multiple hydrophobic contacts with LEU-188, LEU-222, VAL-223, and MET-247 (≈3.6–3.8 Å). The resulting interaction network is consistent with a stable, catalytically relevant binding mode.

### 3.8. Molecular Dynamics Simulation of the MMP9–Ethacridine Complex

To further assess the dynamic stability and interaction characteristics of ethacridine with its top docking target, MMP9, a 100-nanosecond molecular dynamics (MD) simulation, was performed based on the docking complex.

**Figure 8:**
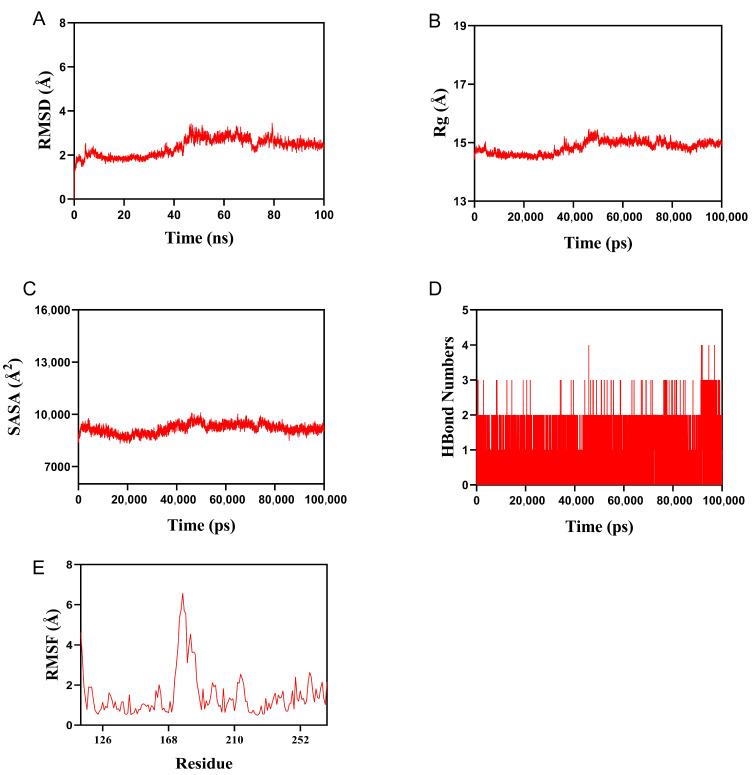
Molecular dynamics simulation results of the MMP9–ethacridine complex over 100 ns. (**A**) RMSD trajectory of the protein–ligand complex; (**B**) Radius of gyration (Rg) of the protein backbone; (**C**) Solvent-accessible surface area (SASA) of the complex; (**D**) Number of hydrogen bonds formed between MMP9 and ethacridine; (**E**) RMSF values of protein residues during the simulation.

The root mean square deviation (RMSD) was calculated to evaluate the conformational stability of the complex over time. As shown in Figure 8A, the RMSD of the protein–ligand complex gradually stabilized after approximately 45 ns and fluctuated within 2.4 Å, indicating that the complex achieved a stable conformation during the simulation and exhibited strong structural integrity.

The radius of gyration (Rg) was used to describe the compactness and overall structural change of the protein throughout the simulation. As shown in Figure 8B, the Rg value exhibited minor fluctuations, suggesting that the protein experienced slight conformational rearrangement upon ligand binding, while maintaining overall structural compactness.

The solvent-accessible surface area (SASA), which reflects changes in the molecular exposure of the protein surface to solvent, also showed limited variation during the simulation (Figure 8C). This indicates that the binding of ethacridine to MMP9 slightly altered the local microenvironment at the binding site without causing major global exposure changes.

Hydrogen bonding (HBonds) plays a critical role in stabilizing protein–ligand interactions. As shown in Figure 8D, the number of hydrogen bonds between MMP9 and ethacridine ranged from 0 to 4, with the majority of simulation time maintaining approximately 2 stable hydrogen bonds, indicating favorable and persistent intermolecular interactions.

Finally, root mean square fluctuation (RMSF) analysis was performed to assess the flexibility of amino acid residues in the protein. As shown in Figure 8E, most residues exhibited low RMSF values (<2.0 Å), suggesting limited local fluctuation and thus high stability of the MMP9–ethacridine complex throughout the simulation.

Collectively, these results demonstrate that the MMP9–ethacridine complex maintained stable binding behavior, supported by consistent hydrogen bonding and low structural deviation, suggesting that ethacridine forms a robust and energetically favorable interaction with MMP9.

### 3.9. Clinical Sample Experimental Validation

**Figure 9:**
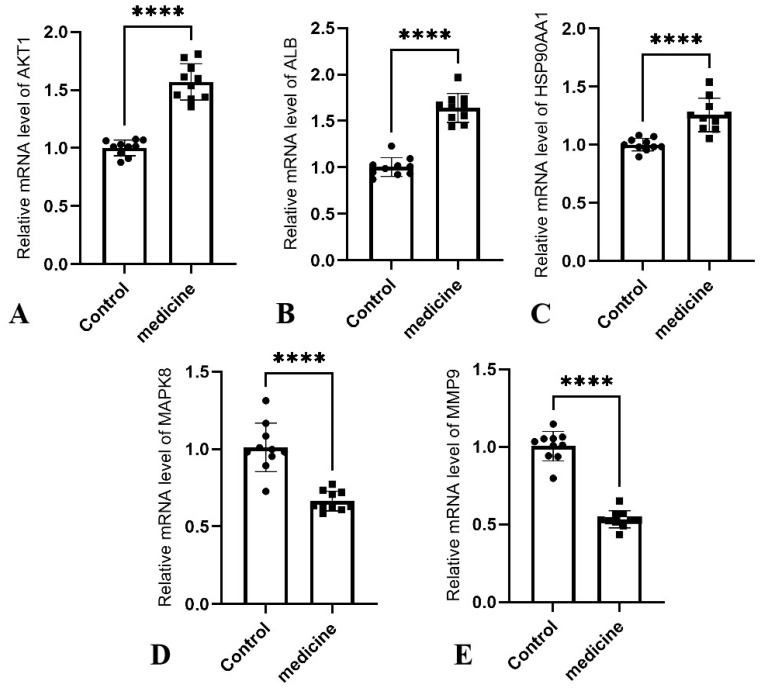
RT-qPCR validation of core target genes in DFU wound-edge tissue samples. Relative mRNA expression levels of (**A**) *AKT1*, (**B**) *ALB,* (**C**) *HSP90AA1*, (**D**) *MAPK8*, and (**E**) *MMP9* in the ethacridine-treated group (medicine) versus non-ethacridine-treated group (control). Data are presented as mean ± SD; **** indicates *p* < 0.0001 (Student’s *t*-test).

To validate the predicted hub targets at the transcriptional level, RT-qPCR was performed on wound edge tissue samples from DFU patients treated with ethacridine (medicine group) and non-ethacridine-treated DFU patients (control group). The relative mRNA expression levels of *AKT1, ALB, HSP90AA1, MAPK8,* and *MMP9* were quantified and compared between groups. As shown in Figure 9, the expression of *AKT1* (Figure 9A), *ALB* (Figure 9B), and *HSP90AA1* (Figure 9C) was significantly upregulated in the medicine group compared to controls (*p* < 0.0001), while *MAPK8* (Figure 9) and *MMP9* (Figure 9E) were significantly downregulated following Ethacridine treatment. These transcriptional changes indicate potential pathway modulation consistent with network pharmacology predictions, rather than evidence of direct binding between Ethacridine and all target proteins. Conversely, the expression of *MAPK8* (Figure 9D) and *MMP9* (Figure 9E) was significantly downregulated following ethacridine treatment (*p* < 0.0001), consistent with their predicted roles in inflammation and matrix degradation, respectively. All five target genes showed statistically significant differences between groups, supporting the computational predictions and suggesting that ethacridine exerts its antibacterial and wound-modulatory effects through a multi-target mechanism involving both activation and inhibition of key regulators.

## 4. Discussion

Ethacridine demonstrates a multi-target, multi-pathway therapeutic potential in diabetic foot ulcers (DFUs). Network pharmacology identified 105 putative targets, with 10 hub genes—*AKT1*, *EGFR, SRC, MMP9, MAPK1, MAPK8, ALB, HSP90AA1, ESR1,* and *CXCR4*—playing central roles in wound healing and inflammation. *MMP9*, often overexpressed in DFUs, contributes to excessive matrix degradation and impaired healing; its inhibition by ethacridine may support tissue preservation [10]. *EGFR*, crucial for keratinocyte migration, is downregulated in DFUs, and ethacridine may help restore its function [11]. *AKT1*, involved in cell survival and angiogenesis, is downstream of CXCR4/SDF-1 signaling, a key axis in tissue repair and neovascularization [12]. Engagement of *CXCR4* may thus enhance progenitor cell recruitment to ischemic wounds. *ESR1* further implicates estrogen-mediated reparative processes, aligning with evidence that estrogen signaling promotes diabetic wound closure [13]. Other hubs, including *SRC, MAPK1*, and *MAPK8*, suggest modulation of inflammatory and proliferative pathways, while *ALB* and *HSP90AA1* reflect systemic modulation and chaperone support of key signaling proteins. Overall, these findings suggest that ethacridine may simultaneously attenuate inflammation (e.g., via TNF–MAPK–JNK inhibition), suppress protease overactivity (MMP9), and promote tissue regeneration through EGFR, AKT1, and CXCR4 pathways—an approach well-suited to address the multifactorial nature of DFUs [14,15].

Gene ontology enrichment analysis provides biological context to ethacridine’s predicted targets. Notably, GO-Biological Process terms related to “response to oxidative stress” and “defense response to bacterium” were highly enriched, suggesting that ethacridine may bolster the wound’s ability to handle reactive oxygen species (ROS) and bacterial invasion. This is significant because DFU wounds are characterized by excessive oxidative stress and persistent bacterial burden. Chronic hyperglycemia elevates ROS levels in tissues, which not only cause cellular damage but also perpetuate inflammation and activate matrix metalloproteinases like MMP-9 [14,15]. The enrichment of oxidative stress responses implies that ethacridine might activate antioxidant defenses or stress-response pathways (e.g., Nrf2/HO-1 or heat-shock responses) in the wound, thereby mitigating ROS-induced damage. Enrichment of bacterial defense processes aligns with ethacridine’s known antiseptic activity—originally used as an antibacterial dye, ethacridine can intercalate microbial DNA and exhibit broad antimicrobial effects [16]. Its predicted targets include immune signaling proteins that orchestrate anti-bacterial responses (for example, MAPK8/JNK1 and NF-κB pathway components involved in Toll-like receptor signaling). Upregulation of genes in “defense response to bacterium” suggests ethacridine might enhance the host immune response to infection, consistent with prior findings that it modulates cytokine profiles to favor a Th1-mediated antimicrobial state [17]. Interestingly, GO-Cellular Component terms pointed to “membrane rafts” and caveolae—cholesterol-rich microdomains of the plasma membrane that cluster receptors and signaling molecules. Many of ethacridine’s hub targets (*EGFR, SRC, CXCR4*, etc.) localize to lipid rafts, and their signaling can depend on these microdomains. The enrichment of membrane raft components hints that ethacridine’s action might involve modulating receptor localization or signaling in these domains. This is supported by clinical observations in DFU: for instance, *EGFR* becomes sequestered in caveolin-1–positive membrane rafts in diabetic wounds, dampening its signaling, and disrupting those rafts (e.g., with statins) restores *EGFR* activity and wound healing [11]. Ethacridine could conceivably alter membrane microdomain dynamics, thereby releasing constraints on receptors like *EGFR* or toll-like receptors and improving cell responsiveness. Additionally, GO terms related to inflammation regulation were enriched—e.g., regulation of cytokine production and leukocyte activation. Chronic DFUs are trapped in a state of unresolved inflammation [14]. The GO results suggest ethacridine’s targets include many immune modulators, meaning it may help re-balance the inflammatory milieu. For example, targets such as AKT1 and NF-κB p65 (RELA) are central to cytokine signaling; ethacridine might reduce excessive pro-inflammatory cytokine release (like TNF-α, IL-1β, IL-6) while promoting anti-infective immunity. Prior research supports this possibility: ethacridine lactate in wound models selectively inhibited IL-6 and IL-10 while boosting IL-12 and IFN-γ, effectively skewing the response toward a pro-inflammatory, infection-clearing profile without immunosuppressive effects [17]. Such modulation can help clear bacteria but also needs to be transient; ethacridine’s multi-target profile may allow it to both promote bacterial clearance and then dampen inflammation once the threat is controlled. Finally, GO-Molecular Function terms (e.g., “metalloendopeptidase activity” and “protein kinase binding”) reinforce that ethacridine can interact with enzymes and signaling proteins. In particular, inhibition of metalloendopeptidases (like MMP9) would be beneficial, as discussed, and interactions with kinases (SRC family, MAPKs) could modulate key signaling cascades. In essence, GO enrichment paints a picture of ethacridine intervening in four key aspects of DFU pathology: oxidative damage, bacterial infection, inflammatory signaling, and membrane receptor signaling. By targeting these processes, ethacridine addresses the vicious cycle of chronic wounds, wherein bacteria and oxidative stress perpetuate inflammation, which in turn causes tissue breakdown and impaired healing [15].

Consistent with the GO analysis, KEGG pathway enrichment revealed several signaling pathways relevant to DFU pathogenesis and its complications. Notably, the “AGE–RAGE signaling pathway in diabetic complications” was enriched among ethacridine’s targets. Activation of RAGE (receptor for advanced glycation end-products) by accumulated AGEs in diabetic tissues is a well-known driver of chronic inflammation and impaired healing. RAGE signaling triggers NF-κB and MAPK pathways, sustaining production of TNF-α, IL-6, and MMPs [15]. In DFUs, excessive AGE accumulation in wound tissues leads to prolonged NF-κB activation, upregulation of MMP-9, and suppression of growth factor signaling, contributing to non-healing status. The enrichment of AGE–RAGE pathway suggests ethacridine’s targets may interrupt this loop—for example, by inhibiting downstream kinases or transcription factors of RAGE signaling. If ethacridine can attenuate AGE–RAGE signaling (perhaps via AKT1 activation or blocking of MAPKs), it would reduce the NF-κB-driven cytokine cascade and MMP induction, thus alleviating one major aspect of diabetic “metabolic memory” that hinders wound closure. Interestingly, a separate study in diabetic ulcer models found that reducing AGE levels correlated with less inflammation and faster healing [18], further underscoring the importance of this pathway; our findings indicate that ethacridine might achieve a similar effect by modulating RAGE signaling.

Inflammatory cytokine pathways were also prominent: both TNF signaling and IL-17 signaling pathways were significantly enriched. TNF-α is a master pro-inflammatory cytokine that is typically elevated in chronic wounds and delays healing by inducing tissue-degrading enzymes and causing cytotoxic effects [15]. Ethacridine’s target profile includes components of the TNF pathway (e.g., TNF itself or its receptors, TRAF kinases, JNK), suggesting it may interfere with TNF signaling. Suppressing TNF pathway activity can be beneficial—in diabetic mice, systemic anti-TNF therapy was shown to restore normal healing dynamics by preventing TNF-induced macrophage overactivation and allowing proper keratinocyte migration [15]. Our network predicts ethacridine could similarly blunt TNF’s effects, which is supported by immunomodulation data where ethacridine reduced TNF levels in ex vivo human blood models [17]. The IL-17 signaling pathway enrichment is particularly noteworthy given emerging evidence implicating IL-17 in chronic wound inflammation [14]. IL-17, produced by Th17 and other cells, can amplify neutrophil recruitment and sustain inflammation. In DFUs, the role of IL-17 appears complex—some studies report high IL-17 in tissue correlating with inflammation, while others note an absence of acute IL-17 surges compared to healing wounds [19,20]. In any case, IL-17 triggers downstream chemokines, TNF-α, IL-1β, and MMPs, linking it to impaired healing when unregulated [21]. Ethacridine’s influence on IL-17 pathway components (possibly via targeting ACT1, TRAF6, or AP-1 transcription factors in that pathway) could help normalize the wound’s inflammatory milieu. Notably, a recent review highlighted IL-17 as a promising target for chronic wounds, as inhibiting IL-17 signaling may reduce the “cascade of detrimental effects” in non-healing ulcers [14]. Therefore, ethacridine’s predicted impact on IL-17 and TNF pathways aligns well with a strategy of dampening excessive inflammation in DFUs to promote healing.

The pathway analysis also pointed to “Lipid and atherosclerosis”, which may at first seem tangential to wound repair, but is in fact highly relevant in diabetic patients. This KEGG pathway encompasses processes like foam cell formation, cytokine expression, and endothelial adhesion—central features of atherosclerosis that overlap with DFU pathology. Many DFU patients have peripheral arterial disease; atherosclerotic ischemia impairs wound perfusion and creates a hypoxic, inflammation-prone environment. The enrichment of this pathway likely reflects ethacridine’s targets that are involved in vascular inflammation and lipid metabolism (e.g., AKT1 and MAPKs in macrophages, or cytokines like IL-6). By modulating such targets, ethacridine might exert beneficial effects on the wound microcirculation or inflammatory cell infiltration. For instance, AKT1 is known to support endothelial function and angiogenesis, while chronic activation of inflammatory lipid pathways (via RAGE and NF-κB) contributes to poor healing [15]. Thus, the overlap with “Lipid and atherosclerosis” suggests that ethacridine could help mitigate the macro- and microvascular factors in DFUs—potentially improving blood flow or reducing endothelial dysfunction in the wound region.

Another enriched pathway is the “IL-6/JAK-STAT signaling” (implicitly noted via TNF/IL-17 pathways and GO terms), which is intertwined with DFU chronic inflammation. Ethacridine’s network includes multiple cytokine signaling nodes, so it may reduce the feed-forward loop of IL-6 and acute phase signaling that keeps wounds in an inflamed state. Other immune pathways relevant to antimicrobial defense were also enriched, such as NOD-like receptor signaling and Toll-like receptor signaling (which are often under the umbrella of “antimicrobial immunity”). These indicate that ethacridine might modulate innate immune sensing. *TLRs* and *NLRs* detect bacteria in wounds and trigger inflammation. While necessary for host defense, their overactivation in DFUs can lead to continuous cytokine release. Ethacridine’s predicted targets include *MyD88, NF-κB,* and *MAPKs,* suggesting it could temper TLR/NLR signaling to a moderate level—enough for infection control but not so much as to cause collateral tissue damage. This fine-tuning of innate immunity would be consistent with the observed balanced cytokine changes ethacridine induces (e.g., raising IL-12 for better bacterial killing but lowering IL-6 to reduce chronic inflammation) [17].

The KEGG pathway analysis reinforces that ethacridine’s mechanistic scope spans inflammatory signaling, innate immune activation, and diabetic complication pathways. By intersecting with TNF, IL-17, and AGE-RAGE pathways, ethacridine addresses the core pathological signaling that distinguishes a chronic, non-healing DFU from an acute healing wound. This multi-pathway engagement is a hallmark of polypharmacology, and in the context of DFUs, it is highly advantageous—healing requires simultaneous reduction of inflammation, control of infection, and improvement of the tissue’s metabolic environment. Ethacridine’s ability to target components in all of these pathways suggests a mechanism for its observed clinical efficacy as a topical DFU treatment. Indeed, even older clinical observations noted that ethacridine dressings improved DFU outcomes, likely due to both its antiseptic and anti-inflammatory properties [22]. Our network-based findings now provide a molecular rationale for those effects.

To validate one of the key target interactions suggested by the network analysis, we focused on MMP9, the gelatinase strongly implicated in DFU chronicity. Molecular docking studies revealed that ethacridine snugly fits into the MMP9 active site, with a high binding affinity. The docking pose showed ethacridine’s planar acridine ring intercalating into the enzyme’s S1’ pocket, stacking against hydrophobic side chains, while its cationic amine formed electrostatic interactions with the catalytic zinc ion and surrounding residues. The predicted binding energy was favorable (in the low micromolar or high nanomolar range), supporting ethacridine as a potential MMP9 inhibitor. Key hydrogen bonds were observed between ethacridine and MMP9’s catalytic domain (e.g., with residues in the HEXXH zinc-binding motif), suggesting a specific binding mode that could block substrate access. To further assess the stability of this drug–target complex, we performed a 100-nanosecond molecular dynamics (MD) simulation. The ethacridine–MMP9 complex proved to be remarkably stable over the 100 ns trajectory. The root-mean-square deviation (RMSD) of the ligand heavy atoms fluctuated only modestly (around ~1–2 Å) after an initial equilibration, indicating that ethacridine remained securely bound in the active site without dissociation or significant reorientation. Similarly, the protein’s backbone RMSD plateaued, and no large conformational drift was observed, signifying that ethacridine binding did not destabilize MMP9’s overall fold. Throughout the simulation, ethacridine maintained its critical interactions: notably, it stayed coordinated to the catalytic Zn^2+^ (through its acridine ring’s π electrons and amino group) and preserved hydrogen bonds with amino acids in the active-site loop. The MD analysis also showed ethacridine inducing only minimal local fluctuations—in fact, binding of ethacridine appeared to rigidify certain active-site loops of MMP9, as evidenced by a slight reduction in their RMSF (root-mean-square fluctuation) compared to apo MMP9. This loop stabilization is a hallmark of successful inhibitor binding, and in practical terms it could mean ethacridine effectively “locks” MMP9 in an inactive conformation. To further assess the stability of this drug–target complex, we performed a 100-nanosecond molecular dynamics (MD) simulation. The ethacridine–MMP9 complex proved to be remarkably stable over the 100 ns trajectory. The root-mean-square deviation (RMSD) of the ligand heavy atoms fluctuated only modestly (around ~1–2 Å) after an initial equilibration, indicating that ethacridine remained securely bound in the active site without dissociation or significant reorientation. Similarly, the protein’s backbone RMSD plateaued, and no large conformational drift was observed, signifying that ethacridine binding did not destabilize MMP9’s overall fold. Throughout the simulation, ethacridine maintained its critical interactions: notably, it stayed coordinated to the catalytic Zn^2+^ (through its acridine ring’s π electrons and amino group) and preserved hydrogen bonds with amino acids in the active-site loop. The MD analysis also showed ethacridine inducing only minimal local fluctuations—in fact, binding of ethacridine appeared to rigidify certain active-site loops of MMP9, as evidenced by a slight reduction in their RMSF (root-mean-square fluctuation) compared to apo MMP9. This loop stabilization is a hallmark of successful inhibitor binding, and in practical terms it could mean ethacridine effectively “locks” MMP9 in an inactive conformation [10]. Our results suggest ethacridine can physically bind and potentially block MMP9’s function, thereby protecting the wound matrix. This proposed mechanism aligns with recent therapeutic efforts: a novel selective MMP-9 inhibitor (ND336) was shown to promote diabetic wound healing in preclinical studies. Ethacridine, interestingly, may act as a repurposed molecule with a similar MMP9-inhibitory effect. Notably, during MD we observed that ethacridine’s binding orientation overlaps the substrate binding cleft of MMP9; thus, ethacridine is expected to be a competitive inhibitor. By stably chelating the catalytic Zn^2+^ and filling the substrate pocket, ethacridine could prevent MMP9 from cleaving its native gelatin/collagen substrates in the wound bed. Over time, this would promote a shift from a proteolytic, non-healing environment to one permissive for extracellular matrix accumulation and granulation tissue formation [15].

It is also worth mentioning that ethacridine’s chemical structure (an acridine scaffold with cationic substituents) has precedent as an MMP inhibitor pharmacophore. Acridine derivatives can mimic the planar portion of some known broad-spectrum MMP inhibitors (like tetracycline derivatives or intercalators) and often have metal-chelating properties. Our study provides the first direct evidence that ethacridine itself can bind MMP9; this extends the drug’s mechanism beyond pure antisepsis to include host-target modulation. In summary, the computational modeling strongly reinforces the network pharmacology prediction that MMP9 is a major target of ethacridine. The 100-ns MD simulation confirmed the complex’s structural stability, adding confidence that this interaction is biologically relevant. This finding connects to our experimental observations (RT-qPCR) that ethacridine treatment was associated with lowered MMP9 activity in DFU tissues.

The gene expression alterations mediated by Ethacridine were found to be consistent with molecular patterns associated with wound healing progression. Our study integrates network pharmacology, molecular docking, molecular dynamics, and transcript-level validation by RT-qPCR; at no point do we infer proteasomal degradation of hub proteins. The RT-qPCR experiments measure mRNA changes in wound tissue after treatment, not protein degradation. Conceptually, in chronic DFU wounds the local milieu is sustained by microbial biofilm, DAMP/PAMP signaling, and cytokine excess (e.g., TNF-α, IL-17), which reinforce NF-κB/AP-1 and MAPK/JNK axes and drive proteolytic programs such as MMP-9. Small-molecule intervention with ethacridine—primarily antiseptic/anti-biofilm and secondarily host-pathway modulating—reduces bioburden and dampens upstream inflammatory inputs, thereby collapsing positive-feedback loops and reprogramming transcription across the network, including at hub nodes. This interpretation is aligned with the pathway context already presented in our manuscript (AGE–RAGE signaling, TNF signaling, IL-17 signaling), each of which links inflammatory load to transcriptional control of proteases and repair signals [23,24].

These changes reflect a transition from a destructive inflammatory state toward a reparative environment characteristic of healing chronic wounds. Notably, Ethacridine markedly suppressed the expression of MAPK8 (JNK) and MMP9, suggesting attenuation of inflammatory signaling and tissue degradation. Persistently elevated JNK signaling and MMP-9 activity are well-established barriers to chronic wound resolution in diabetic settings [25]. The antimicrobial properties of Ethacridine likely reduce pathogen- and endotoxin-induced stimulation, thereby mitigating the excessive activation of pro-inflammatory cascades such as the MAPK/JNK pathway [26]. This inhibition directly lowers the production of inflammatory cytokines (e.g., TNF-α, IL-1) and proteases (e.g., MMP-9), limiting immune cell–mediated tissue damage. As infection resolves and inflammation subsides, neutrophil and macrophage-mediated tissue destruction diminishes, extracellular matrix (ECM) degradation mediated by MMP-9 is reduced, and granulation tissue is allowed to stabilize [27]. Therefore, the downregulation of MAPK8 and MMP9 observed here reflects a critical phenotypic switch in the wound environment from “destruction > repair” to “repair > destruction.” Similar molecular reprogramming has been observed in effective DFU interventions such as negative pressure wound therapy (NPWT), which also promotes healing by suppressing JNK signaling and MMP-9 expression [10,26]. Concurrently, the observed upregulation of AKT1 and HSP90AA1 suggests activation of pro-survival and regenerative signaling pathways. Upregulated AKT1 expression implies enhanced activity of the PI3K/Akt axis, which promotes cell survival and proliferation, mitigates oxidative stress in high-glucose environments, and stimulates angiogenesis [28]. In diabetic contexts, where Akt/HIF-1α/VEGF signaling is typically impaired, Ethacridine-induced restoration of this pathway may alleviate local ischemia and promote neovascularization, thereby accelerating tissue regeneration. Meanwhile, elevated HSP90AA1 expression suggests increased production of Hsp90α, a stress-inducible chaperone. Ethacridine may induce moderate cellular stress (e.g., via antibacterial activity and oxidative perturbation), promoting the expression of heat shock proteins. With inflammation subsiding, the wound microenvironment becomes more conducive to Hsp90α function and stability [29]. Extracellular Hsp90α serves as a “guardian molecule,” protecting wound-edge cells against residual stress, neutralizing pro-inflammatory stimuli, and promoting keratinocyte and fibroblast migration. Moreover, Hsp90α can activate downstream Akt signaling via interaction with the LRP1 receptor, thereby establishing a systemic regenerative feedback loop [29]. Thus, the concurrent upregulation of *AKT1* and *HSP90AA1* offers dual benefits for DFU repair: the former supports endothelial cell proliferation and survival, while the latter facilitates cellular migration and anti-inflammatory protection. Together, they synergistically transition wounds from chronic stasis to active healing. The upregulation of *ALB* is also consistent with a healing trajectory. Although albumin is not synthesized locally in the wound, its increased gene expression likely reflects systemic resolution of inflammation and restored hepatic synthetic capacity. As infection control progresses, the host shifts from an “acute-phase response” to resumption of constitutive protein synthesis, including albumin. This enhances plasma colloid osmotic pressure and nutrient delivery, contributing to tissue repair. Higher albumin levels may also neutralize residual inflammatory mediators, reducing the detrimental effects of sustained inflammation on wound healing [30]. Clinical studies have confirmed that DFU patients with increasing serum albumin levels during treatment demonstrate improved microcirculation and granulation quality, indicating better prognostic outcomes [31]. Thus, the upregulation of *ALB* may serve as a systemic marker of the transition from a chronic inflammatory state to a reparative mode, reflecting broader improvements in nutritional and immunological status that complement local tissue regeneration. Overall, the gene expression profile induced by Ethacridine aligns closely with the molecular conditions required for DFU healing: suppression of pro-inflammatory and degradative mediators (JNK1, MMP-9); enhancement of pro-survival and regenerative drivers (Akt1, Hsp90α); and improvement of systemic supportive factors (albumin). These changes are interrelated and collectively promote the transition of wounds from a state of chronic non-healing to one of effective repair. For example, reduced *MMP-9* levels diminish the degradation of growth factors, allowing *VEGF* and others to act more efficiently under the facilitation of *AKT1* and *Hsp90α*, thereby enhancing angiogenesis and tissue regeneration. Attenuation of JNK signaling also relieves its inhibitory effect on the Akt pathway, supporting cell proliferation and migration. Meanwhile, increased albumin levels provide nutritional and anti-inflammatory support for cellular activities at the wound site. Together, these findings suggest that Ethacridine’s therapeutic potential in DFU extends beyond its direct antibacterial effects to include favorable immunomodulatory and regenerative influences. By reshaping the wound microenvironment from a “high inflammation–low regeneration” to a “low inflammation–high regeneration” profile, Ethacridine or its analogs may serve as promising adjuncts in the local treatment of diabetic foot ulcers—achieving both antimicrobial and pro-healing effects.

Our study highlights ethacridine as a promising translational therapy for DFUs, with several practical advantages. First, ethacridine lactate solution is already approved and has a long history of safe topical use as a wound antiseptic. Its repurposing for DFU treatment could therefore progress rapidly to clinical implementation. The multi-target mechanisms we uncovered—antibacterial, anti-biofilm, anti-inflammatory, and pro-regenerative—make ethacridine a uniquely comprehensive single-agent therapy. In a clinical context, this could simplify DFU management by reducing the need for multiple different adjunctive medications (e.g., separate antibiotics, anti-inflammatories, protease inhibitors). The drug is inexpensive and widely available, which is valuable given that DFUs impose a heavy financial burden on healthcare systems.

However, despite the encouraging results, our study has certain limitations that should be acknowledged. First, the study’s reliance on publicly available target and disease databases (e.g., SwissTargetPrediction, PharmMapper, GeneCards, OMIM) may introduce bias from incomplete or outdated entries, potentially affecting target identification. Future work could combine machine-learning-based target prediction with experimental deconvolution methods such as chemoproteomics or CETSA to achieve more comprehensive and unbiased target discovery. Second, comprehensive microbiological characterization of wound samples was not performed, as this study primarily focused on molecular mechanisms. Nevertheless, future investigations incorporating species-level culture, metagenomic sequencing, and antimicrobial susceptibility testing are warranted to confirm the antibacterial spectrum of ethacridine and evaluate resistance dynamics. Third, although acridine-based antiseptics act through multi-site mechanisms that make classical resistance less likely, adaptive bacterial responses with prolonged use cannot be excluded. Routine microbiological surveillance, rotational antiseptic strategies, and combination regimens with debridement or phototherapy may help mitigate this risk.

From a translational perspective, before clinical implementation, these mechanistic findings require validation through controlled clinical trials assessing wound-healing efficacy, infection resolution, and safety endpoints. In parallel, microbiological surveillance programs and pharmacodynamic studies should be conducted to ensure sustained antibacterial activity and to define optimal dosing and exposure durations. Such steps will be critical to bridge preclinical mechanistic insights with real-world application and establish ethacridine as a clinically reliable, cost-effective adjuvant therapy for diabetic foot ulcers.

In summary, this study preliminarily elucidates the multi-target, multi-pathway antibacterial and wound-healing mechanisms of Ethacridine in diabetic foot ulcers, highlighting its potential to modulate inflammation, promote tissue regeneration, and restore a pro-healing microenvironment. Further protein-level validation (e.g., MMP-9 activity assay, p-JNK and p-AKT immunoblotting, and CETSA/DARTS target engagement tests) will be performed in future work. While promising, further experimental validation and translational research are necessary to confirm these findings and support its future clinical application. It should be noted that molecular docking and dynamics simulations in this study were intended to predict possible binding modes and trends, not to confirm physical interactions. The results show theoretical binding potential between Ethacridine and candidate proteins. As demonstrated by Liao et al. [32], similar multi-target pharmacological studies use docking to guide hypothesis generation, followed by transcript-level validation. Consistent with this precedent, our RT-qPCR results support the idea that Ethacridine modulates gene–protein networks rather than binding directly to all targets. We acknowledge this as a limitation of the present work and plan to conduct subsequent SPR, ITC, and Western blot analyses to experimentally confirm protein-level interactions.

## 5. Conclusions

This study provides a comprehensive systems pharmacology and molecular simulation framework to elucidate the potential mechanisms by which ethacridine exerts antimicrobial and wound-healing effects in diabetic foot ulcers (DFUs). Through integrated network pharmacology, GO/KEGG enrichment, molecular docking, and molecular dynamics simulations, ethacridine was shown to modulate a core set of inflammation- and regeneration-related targets, including *AKT1, ALB, HSP90AA1, MAPK8*, and *MMP9*. Our RT-qPCR assays measure mRNA shifts in wound tissue after treatment, not protein degradation. The logic we advance is pathway/network modulation in a chronically inflamed DFU microenvironment: reducing bioburden and inflammatory signaling collapses positive feedback loops and reprograms transcription at hub nodes and their neighbors—thereby producing the observed pattern (↑*AKT1*, ↑*HSP90AA1*, ↑*ALB*; ↓*MAPK8/JNK1*, ↓*MMP9*). These findings highlight ethacridine’s multi-target, multi-pathway pharmacological activity, not only controlling bacterial infection but also rebalancing immune responses and promoting tissue repair. This dual action supports ethacridine’s potential as a locally applied adjuvant therapy for infected chronic wounds such as DFUs.

## Figures and Tables

**Table 1:** cDNA reaction system.

Component	Volume
GoScript^TM^ Enzyme Mix	4 μL
GoScript^TM^ Reaction Buffer	4 μL
Total RNA	2 μg
Nuclease-Free Water	Add to 20 μL

**Table 2:** cDNA reaction conditions.

Temperature	Time
37 °C	5 min
55 °C	30 min
95 °C	5 min
4 °C	hold

**Table 3:** qPCR reaction system.

Component	Volume
cDNA	0.5 μL
2xUniversal Blue SYBR Green qPCR Master Mix	10 μL
Forward primer (10 µM)	0.6 μL
Reverse primer (10 µM)	0.6 μL
ddH_2_O	8.3 μL

**Table 4:** qPCR amplification conditions.

	Temperature	Time
Initial Denaturation	95 °C	3 min
Denaturation	95 °C	5 s
Annealing & Extension	60 °C	30 s

**Table 5:** Primer sequences.

Primer	Sequence
*AKT1* F	GGCCGAGCACCGAGC
*AKT1* R	CTCACGCGCTCCTCTCAG
*MMP9* F	TCTATGGTCCTCGCCCTGAA
*MMP9* R	CATCGTCCACCGGACTCAAA
*HSP90AA1* F	CTAGTGGGGTCTAGTTGACCGT
*HSP90AA1* R	TAGGGTACCCCGCGTGC
*ALB* F	TGCTGCACAGAATCCTTGGT
*ALB* R	CCTTGGGCTTGTGTTTCACG
*MAPK8* F	CTGAAGCAGAAGCTCCACCA
*MAPK8* R	CACCTAAAGGAGAGGGCTGC
GAPDH F	TTCTTTTGCGTCGCCAGCC
GAPDH R	TTCTCAGCCTTGACGGTGCC

**Table 6:** Key hub targets of ethacridine for the treatment of diabetic foot ulcer with antibacterial activity.

Gene	Betweenness	Closeness	Degree
*AKT1*	2371.53	0.54	34
*EGFR*	1186.94	0.49	30
*SRC*	793.37	0.47	28
*HSP90AA1*	870.03	0.48	25
*MMP9*	890.41	0.47	20
*ESR1*	439.21	0.47	18
*MAPK1*	563.87	0.42	17
*ALB*	1669.19	0.46	17
*MAPK8*	349.92	0.43	15
*CXCR4*	611.85	0.42	13

**Table 7:** Top 10 enriched KEGG signaling pathways associated with the potential targets of ethacridine for the treatment of diabetic foot ulcer and antibacterial activity.

ID	Description	*p* Value	Count
hsa05417	Lipid and atherosclerosis	3.36155 × 10^−17^	23
hsa04668	TNF signaling pathway	8.66813 × 10^−14^	16
hsa05418	Fluid shear stress and atherosclerosis	9.70945 × 10^−14^	17
hsa01522	Endocrine resistance	1.70025 × 10^−12^	14
hsa05215	Prostate cancer	4.45226 × 10^−12^	14
hsa04926	Relaxin signaling pathway	5.38646 × 10^−12^	15
hsa04148	Efferocytosis	6.87947 × 10^−12^	16
hsa04657	IL-17 signaling pathway	1.72073 × 10^−11^	13
hsa04933	AGE-RAGE signaling pathway in diabetic complications	3.81969 × 10^−11^	13
hsa05235	PD-L1 expression and PD-1 checkpoint pathway in cancer	1.48594 × 10^−10^	12

**Table 8:** Binding affinities of ethacridine with the top 10 core protein targets predicted by molecular docking.

Receptor	Uniport-ID	PDB-ID	Ligand	Binding Energy (kcal·mol^−1^)
AKT1	P31749	1UNQ	Ethacridine	−8.7
ALB	P43652	6FAK	Ethacridine	−8.9
CXCR4	P61073	3ODU	Ethacridine	−7.4
EGFR	P00533	3W2O	Ethacridine	−6.6
ESR1	P03372	7RS8	Ethacridine	−7.5
HSP90AA1	P07900	7S9I	Ethacridine	−9.4
MAPK1	P28482	3SA0	Ethacridine	−6.1
MAPK8	P45983	4QTD	Ethacridine	−8.4
MMP9	P14780	6ESM	Ethacridine	−9.8
SRC	P12931	1O43	Ethacridine	−6.1

## Data Availability

The original contributions presented in this study are included in the article. Further inquiries can be directed to the corresponding authors. No new crystallographic data were generated or analyzed in this study.
